# *In silico* drug absorption tract: An agent-based biomimetic model for human oral drug absorption

**DOI:** 10.1371/journal.pone.0203361

**Published:** 2018-08-31

**Authors:** Jianyuan Deng, Anika Jhandey, Xiao Zhu, Zhibo Yang, Kin Fu Patrick Yik, Zhong Zuo, Tai Ning Lam

**Affiliations:** 1 School of Pharmacy, Faculty of Medicine, The Chinese University of Hong Kong, Hong Kong, Hong Kong; 2 School of Pharmacy, University of Nottingham, Nottingham, United Kingdom; 3 Department of Computer Science, Stony Brook University, Stony Brook, NY, United States of America; Universidade Federal do Rio de Janeiro, BRAZIL

## Abstract

**Background:**

An agent-based modeling approach has been suggested as an alternative to traditional, equation-based modeling methods for describing oral drug absorption. It enables researchers to gain a better understanding of the pharmacokinetic (PK) mechanisms of a drug. This project demonstrates that a biomimetic agent-based model can adequately describe the absorption and disposition kinetics both of midazolam and clonazepam.

**Methods:**

An agent-based biomimetic model, *in silico* drug absorption tract (ISDAT), was built to mimic oral drug absorption in humans. The model consisted of distinct spaces, membranes, and metabolic enzymes, and it was altogether representative of human physiology relating to oral drug absorption. Simulated experiments were run with the model, and the results were compared to the referent data from clinical equivalence trials. Acceptable similarity was verified by pre-specified criteria, which included 1) qualitative visual matching between the clinical and simulated concentration-time profiles, 2) quantitative similarity indices, namely, weighted root mean squared error (RMSE), and weighted mean absolute percentage error (MAPE) and 3) descriptive similarity which requires less than 25% difference between key PK parameters calculated by the clinical and the simulated concentration-time profiles. The model and its parameters were iteratively refined until all similarity criteria were met. Furthermore, simulated PK experiments were conducted to predict bioavailability (F). For better visualization, a graphical user interface for the model was developed and a video is available in Supporting Information.

**Results:**

Simulation results satisfied all three levels of similarity criteria for both drugs. The weighted RMSE was 0.51 and 0.92, and the weighted MAPE was 5.99% and 8.43% for midazolam and clonazepam, respectively. Calculated PK parameter values, including area under the curve (AUC), peak plasma drug concentration (C_max_), time to reach C_max_ (T_max_), terminal elimination rate constant (Kel), terminal elimination half life (T_1/2_), apparent oral clearance (CL/F), and apparent volume of distribution (V/F), were reasonable compared to the referent values. The predicted absolute oral bioavailability (F) was 44% for midazolam (literature reported value, 31–72%) and 93% (literature reported value, ≥ 90%) for clonazepam.

**Conclusion:**

The ISDAT met all the pre-specified similarity criteria for both midazolam and clonazepam, and demonstrated its ability to describe absorption kinetics of both drugs. Therefore, the validated ISDAT can be a promising platform for further research into the use of similar *in silico* models for drug absorption kinetics.

## Introduction

Oral administration is the most popular and accepted route of delivery for medical drugs. Successful oral therapeutics must pass through the gastrointestinal (GI) barrier to reach systemic circulation, where the drug is distributed to its site of action. Within the GI tract, numerous physiological processes influence the rate and extent of drug absorption. Hence, understanding the mechanisms underlying bioavailability (F) is essential to the development of pharmaceutical products intended for oral delivery.

**Current models for drug absorption: physiologically based pharmacokinetic models.** Researchers have devised numerous increasingly complex models to describe all the aspects of PK and pharmacodynamic (PD) phenomena. One approach is *in vitro–in vivo* extrapolation by using physiologically based pharmacokinetic (PBPK) models that represent human intestinal drug absorption. A system of ordinary differential equations is derived to describe the movement of drugs along the GI segments; each is represented by a PK compartment. Landersdorfer and Jusko [1] have summarized many features of these so-called ‘mechanism-based’ models.

However, inter-individual variability, *e*.*g*., individual enzyme expression levels or variants, spatial heterogeneity, *e*.*g*., changing luminal environment, and irregular temporal dynamics, *e*.*g*., food intake, are difficult to be described by smooth ordinary differential equations in common PBPK models. Mechanisms, variabilities and uncertainties may be determinants of (individual) bioavailability and bioequivalence, and therefore, an alternative to existing PBPK models may better represent them [2].

**Synthetic, agent-based biomimetic modeling.** Novel research techniques and computational tools to achieve a deeper insight [3] into the mechanisms which are responsible for intra- and inter-individual differences in bioavailability data are badly needed [4]. Recently, synthetic, agent-based models have been suggested as a novel approach in biomedical modeling and simulation. Agent-based models (ABMs) employ an object-oriented, discrete-event modeling technique centered on the behaviors and interactions of the individual autonomous components of a system. ABMs focus on the agents’ rules and local interactions between individual components and their environment, thereby generating a ‘virtual world’ in which *in silico* experiments are executed. ABMs are well suited to translate existing biomedical ontologies into a dynamic model, and, as such, an ABM is essentially a knowledge repository for the observations generated in *in vitro*, *in vivo*, or clinical experiments [5].

Unlike how inductive PBPK models are constructed, in ABM, abstract biomimetic software components are assembled to form a computational analogue of the referent biological system [6–9]. A concrete mapping exists between *in silico* components and assembly, and between corresponding micro-anatomic and physiological details at corresponding levels and scales. Hence, the design of the components is guided by current knowledge and hypotheses. As recommended by Hunt *et al*. [8], relational grounding will be employed for model internal consistency and transparent knowledge representation. Besides pharmaceutical research, ABMs have been used in complex multi-attribute biomedical problems, including sepsis [10], systemic inflammation [11], cystogenesis [12], leucocyte activation and dynamics [13,14], and cancer [15–17]. Hunt *et al*. [6,18] and Cosgrove *et al*. [19] have summarized the key concepts and advantages of agent-based models.

**Objectives.** In this project, we aimed to develop an *in silico* agent-based analogue of the human GI tract to model oral drug absorption. Assembling with abstract semi-validated prototypical components from previous studies [7,9,20], the model consists of agents that represent the key aspects of the physicochemical and PK properties of the simulated drugs, and the GI physiological features. They are validated against available human absorption data from bioequivalence studies for two drugs, namely, midazolam and clonazepam.

## Materials and methods

### Clinical bioequivalence study

#### Subjects and study protocol

The bioequivalence studies were conducted under study protocol approved by the Joint Clinical Research Ethics Committee of The Chinese University of Hong Kong and New Territories East Cluster (CUHK-NTEC). All subjects were non-smoking subjects and were treated at The Prince of Wales Hospital, Hong Kong in accordance with current Good Clinical Practices between August 2008 and July 2009. Written informed consent was obtained from all subjects before initiation of these two studies. The data from two clinical bioequivalence studies on midazolam and clonazepam provide the referent data to which comparisons are made. These two drugs were chosen because of availability of densely sampled data, and the marked differences in their concentration-time profiles. Separate approval from the clinical research ethics committee was obtained to access and analyze the archived data.

The referent products used in the clinical studies were Dormicum (midazolam) 15 mg tablets and Rivotril (clonazepam) 2 mg tablets. Each bioequivalence study was a two-treatment, two-period, two-sequence crossover design involving 15 (midazolam) or 14 (clonazepam) subjects (one subject is excluded from the data analyses for taking alcohol).

#### Blood collection and sample assay

Following the oral administration of the drug, blood samples (approximately 5 mL of blood per sample) were taken at 0, 0.5, 1, 1.5, 2, 3, 4, 6, 8, 10, 12, and 24 hours post drug administration for both studies. In addition, two additional time points for midazolam (0.25, 2.5 hours) and three additional samples for clonazepam (48, 72, 96 hours) were taken. All blood samples were centrifuged immediately after collection and separated plasma samples were stored at -80°C until assay.

Plasma concentrations of midazolam and clonazepam were determined by validated Liquid Chromatography-Mass Spectrometry (LC-MS-MS) methods with respect to precision, accuracy, specificity and sensitivity before application. Quality control samples for midazolam were at concentrations of 2.5, 25 and 200 ng/mL and 1.6, 10 and 40 ng/mL for clonazepam, which were utilized for assay validation. The LC-MS-MS system consisted of a Perkin-Elmer liquid chromatography and Q-Trap mass spectrometer (Perkin-Elmer Norwalk, CT, USA). Chromatography was carried out using a Waters XBridge C18 column (4.6 mm × 250 mm, 5 μm), which was proceeded with a Waters XBridge C18 guard column (4.6 × 20 mm, 5 μm). The mobile phase consisted of 10 mM ammonia acetate and acetonitrile, run by a gradient program. The electrospray ionization for midazolam was performed in the positive mode, with multiple reaction monitoring (MRM) of m/z 326→291 for midazolam and 301→255 for internal standard temazepam. Meanwhile, for clonazepam, the electrospray ionization was performed in the negative mode, with multiple reaction monitoring (MRM) of m/z 314→278 for clonazepam and 269→241 for internal standard nordazepam. The detailed assay method is also described in reference [21].

### Synthetic, agent-based biomimetic modeling

The objective of the current study was to construct a synthetic, agent-based, biomimetic model that can reproduce, and therefore help to explain, the observed absorption and disposition phenomena for midazolam and clonazepam. The successfully validated model would then serve as an executable knowledge embodiment about their PKs [6,7,19]. Briefly, the modeling approach was as follows. Taking the multi-scale, middle-out approach, we started by designing and developing biomimetic components that represent biological entities involved in human GI drug absorption. We then assembled and connected these components into a larger *in silico* experimental system that is analogous to the human GI tract in a context of a bioavailability PK study. We also identified a set of targeted attributes based on observations from the referent experiment and sought to reproduce these targeted attributes by execution of the model, i.e., simulating the experiment. We adopted relational grounding in parameterizing our model [7,8] and iteratively refined the model by adjusting the model parameters, and changing the rules and logic of the components and their interactions, until pre-specified similarity criteria were met. In other words, the successful model can produce observations that are analogous to, and statistically indistinguishable from, those from the referent experiment. Therefore, the model stands as a plausible mechanism-based explanation of the observed PK phenomena.

#### Components, events and rules

To avoid confusion between *in vitro* or *in vivo* biology and its *in silico* counterparts, smallcaps are used when referring to components and processes in the *in silico* systems, and *italics* are used for their parameters.

**Fig 1:**
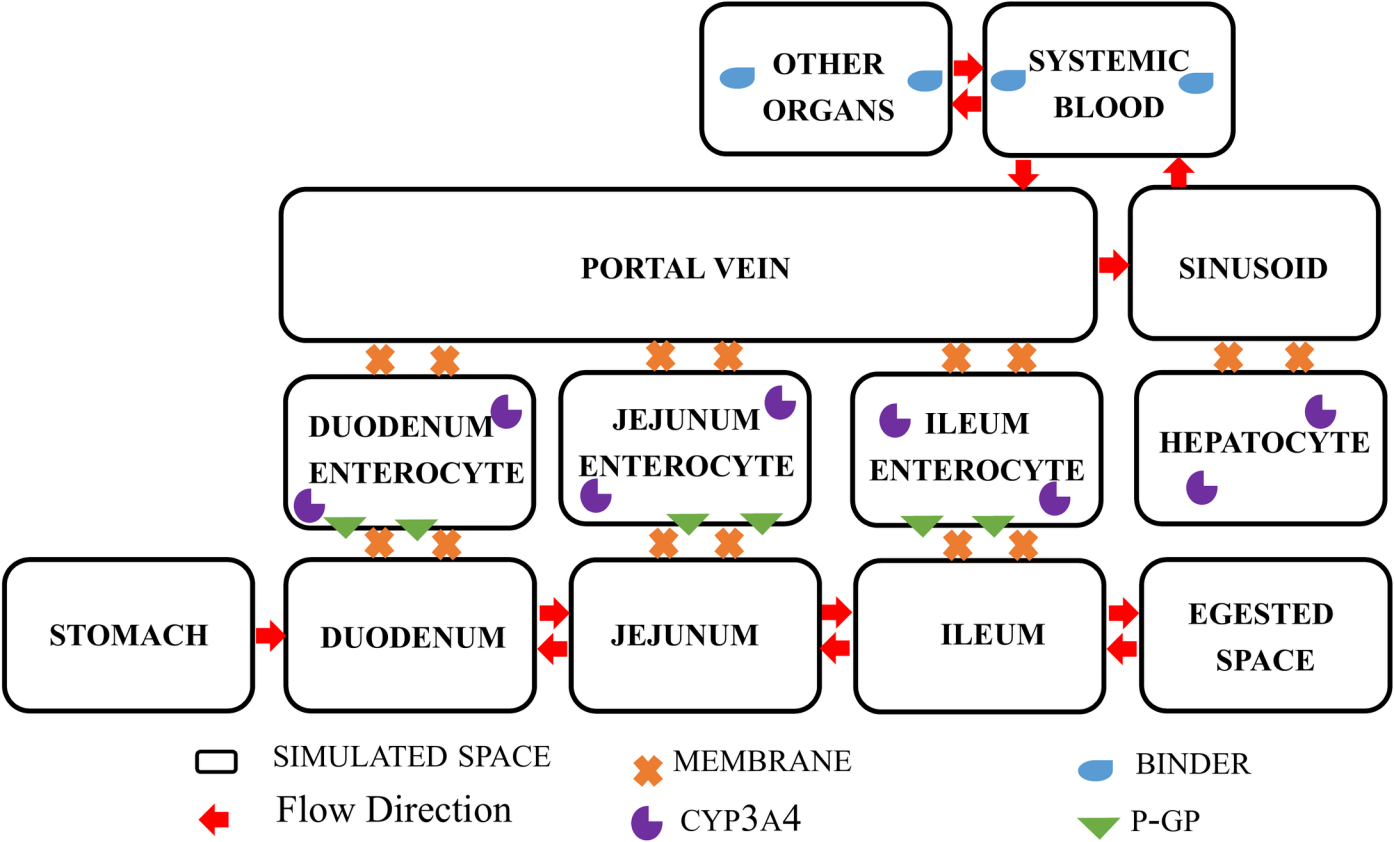
Model structure in two-dimensional schematic.

**Spaces** **and** **membranes.** Spaces are three-dimensional grids that map to referent gastric, intestinal, hepatic and blood spaces. Membranes are two-dimensional grids that represent the physical permeation barriers between spaces. Spaces and membranes are assembled to form the structure of ISDAT; into each space and membrane, components representing drugs, enzymes, transporters, and binders are added. The spaces and membranes used in the ISDAT are shown in Fig 1.

**Drug** **objects and their movement.** Drug objects are autonomous [13] and independent/heterogeneous [20,22] agents. Four types of drug objects were used: midazolam; clonazepam; and one metabolite for each. Each drug object was given properties that reflects its physicochemical and PK properties. The physicochecmical properties include: *molecularWeight* (determine its movement speed); *logP* (determine its innate membrane permeability); and *pKa* (determine its ionization status). The PK properties include the drug’s substrate specificity (both are substrates of CYP3A4), *affinity* to enzymes and *affinity* to binders. Within a space, a drug object moves randomly in all directions. Directional flow (for example, movement of the luminal content) is implemented by two processes: firstly, within each space, drug objects move in a biased random walk and secondly, between connected spaces, a fraction of objects on the edge of the origin space will flow to the adjacent destination space. For the transition between the membrane-separated spaces, when a drug object is near a membrane interface, the drug is given an opportunity to cross–permeate–the membrane and to enter the space on the other side of the membrane. This passive permeation process is probabilistic, and this probability parameter is chosen in respect of the partition equilibrium, which is in turn determined by the drug’s innate permeability and ionization status. A more detailed description of the implementation was presented in our previous reports [7,9,20].

**Enzymes**, **transporters** **and** **binders**. *In silico* enzyme objects (cyp) are placed in enterocytes and hepatocyte to represent the metabolic capacities of these cells. Drug transporters are placed on an apical membrane of enterocytes and they facilitate the efflux of drugs across this membrane. However, the transporters are functionally unnecessary in this study because both midazolam and clonazepam are highly permeable. Binders are placed in systemic blood and other organs to represent the nonspecific binding capacity in the blood and other organs. To be noted, drug elimination is modeled as metabolism by cyp and thus renal excretion of the parent drugs or the metabolites is not modeled.

Drug metabolism in the ISDAT is a three-step process. Firstly, when a drug is in the neighborhood of the enzyme, it may bind to the enzyme. Binding is a probabilistic process characterized by the parameter *affinity*. Secondly, once bound, the enzyme may convert the parent drug into its metabolite. This metabolic step is also probabilistic, and is governed by the parameter *metabolizeProb* of the enzyme. Finally, regardless of whether metabolism has happened or not, the bound drug (or metabolite) may be released from the enzyme, a probabilistic process controlled by the parameter *releaseProb*. The logic of binders is similar, with the exception that a binder may not metabolize its substrate (*i*.*e*., *metabolizeProb* = 0 for binders). More details on the implementation of enzymes, transporters, and binders were presented in our previous reports [7,9].

In summary, simulation with the ISDAT is fundamentally different from the traditional mathematical equation-based PBPK models. In ISDAT, simulation advances in time steps, spaces are represented as discrete space, and processes and interactions between components are represented as events. (Whereas, in the PBPK models, compartments are thought to be continuous and homogenous, movement and processes are expressed as rate constants, and time is continuous.)

### Simulated bioavailability experiment

The assembled ISDAT served as an experimental device; executing it simulated an *in silico* bioavailability experiment. Briefly, the analogue consisted of five spaces corresponding to the GI tract: stomach, duodenum, jejunum, ileum, and colon/egested space—where the drug can longer be absorbed. Adjacent to the small intestinal lumen spaces are the enterocytes spaces which line the intestinal lumen. At the start of the experiment, drug objects were put in the stomach space, representing oral drug administration. Then, they moved along the gi tract, got absorbed via the enterocytes and entered the portal vein. After that, they passed through the sinusoid, which are lined by hepatocytes, where a portion was extracted by the hepatocyte before arriving at the systemic blood. From there, they recycled to the portal vein and sinusoid for further extraction, or distributed to other organs where binders are present.

The experiments were run for 2,500 steps (mapped to 25 hours) for midazolam and 10,000 steps (mapped to 100 hours) for clonazepam. Each experiment was repeated for 15 or 14 times according to test subject numbers for each drug to simulate stochastic variability from run to run. Supporting simulation framework and graphical user interface are also built for visualization purposes (S1 Video). At selected steps, corresponding to the time points in the clinical studies, we took measurements of the drug amount in the systemic blood, simulating measurement of drug levels in the blood.

#### Smoothing procedure

Due to inherent stochasticity in each step, measurements at one single step is highly variable. Therefore, a smoothing procedure is adopted to mitigate the step-to-step variation, which ultimately generate simulation results with more precision.

For each individual simulation, the smoothed amount of drug at a specific step (except for Step 0) is calculated using the following equation

$$Smoothed\;Amount\;at\;Step\;S=\;\frac{1}{2N+1}\;\times \;\sum_{i=S-N}^{S+N}Amount\;at\;Step\;i$$ (1)

where S denotes the intended measurement step and N denotes the interval for smoothing. In the current study, N is 10 (steps). The smoothed amount at each specific step for each individual is then used for the calculation of simulated mean values.

#### Targeted attributes, similarity criteria and iterative refinement

The primary targeted attribute was the mean concentration-time profiles from the two clinical bioequivalence studies. The time course of the mean amount of drug in the systemic blood in ISDAT is mapped to the concentration-time profiles by using two parameters, namely, the *TimeScale* and the *MeasureScale*. Ideally, when all the mean concentration values of simulated profiles at each sampling time point locate within the interval, i.e., mean ±1 SD of the referent clinical profiles, then it is regarded as meeting the first level of similarity, which is to ensure the visual matching.

In addition to visual inspection on the simulated profiles, the similarity between the mean concentration values from simulated experiments and the clinical studies was also quantified to the second level by using two similarity indices: weighted RMSE, and weighted MAPE,

$$weighted\;RMSE=\frac{1}{\sqrt{n}}\sqrt{\sum_{n}\frac{1}{s^{2}}{\left(Clinical\;mean\;value-Simulated\;mean\;value\right)}^{2}}$$ (2)

$$weighted\;MAPE=\frac{1}{n}\sum_{n}\frac{1}{s}\left|\frac{Clinical\;mean\;value-Simulated\;mean\;value}{Clinical\;mean\;value}\right|\times \;100\%$$ (3)

where *n* denotes the repeat times of simulation (and corresponding test subject numbers) and *s* denotes the observed standard deviation (SD) in the clinical data. In other words, the weights used were the SD^2^ and SD for RMSE and MAPE, respectively. The acceptable similarity thresholds for the weighted RMSE and the weighted MAPE are 2.5 and 33%, respectively. These thresholds were selected based on the observed variation in the referent clinical data.

Next, the key PK parameters from both the clinical profiles and the smoothed simulated profiles were calculated for the third level of summary descriptive similarity. These PK parameters included area under the curve (AUC), peak plasma drug concentration (C_max_), time to reach C_max_ (T_max_), terminal elimination rate constant (Kel), terminal elimination half life (T_1/2_), apparent oral clearance (CL/F), and apparent volume of distribution (V/F), by using noncompartmental analysis. The acceptable similarity criterion was less than 25% difference between the clinical and the simulated values for each PK parameter.

The ISDAT was iteratively refined by changing a small subset of model parameters, including the *affinities* of the drugs, and the *expression levels* of enzymes and binders, until achieving the similarity criteria specified above. The system parameters were held to be the same for both drugs, and their relative magnitudes were referred to known physiology, whereas the physicochemical properties parameters for both drugs were fixed to the literature reported values. The iterative refine protocol is detailed in reference [7].

#### Predicted bioavailability

After the ISDAT was parameterized to meet all the above similarity criteria, simulated PK experiments were conducted to predict bioavailability.

Instead of dosing the drug in the stomach space, we started the *in silico* PK experiment with the dose directly in the systemic blood, thus simulating an intravenous dose. The AUC value was calculated, and the absolute bioavailability was calculated by

$$F=\frac{AUC_{oral\;dose}}{AUC_{intravenous\;dose}}\;\times \;100\%$$ (4)

In addition, determinants of oral bioavailability can be described mathematically by the following equation

$$F=F_{a}\;\times \;F_{g}\;\times \;F_{h}$$ (5)

where F_a_ is the fraction of the dose that is absorbed from the intestinal lumen to the intestinal enterocytes; F_g_ is the fraction of the dose that escapes pre-systemic intestinal first pass elimination; and F_h_ is the fraction of the dose that passes through the liver and escapes pre-systemic hepatic first-pass elimination [23]. F_a_ × F_g_ can then be estimated by comparing AUCs when the compound is given orally and via a cannulated hepatic portal vein; similarly, F_h_ can be estimated by comparing AUCs when the compound is given via hepatic portal vein directly and intravenously.

$$F_{a}\;\times \;F_{g}=\frac{AUC_{oral\;dose}}{AUC_{hepatic\;portal\;vein\;dose}}\;\times \;100\%$$ (6)

$$F_{h}=\frac{AUC_{hepatic\;portal\;vein\;dose}}{AUC_{intravenous\;dose}}\;\times \;100\%$$ (7)

The ISDAT-predicted F, F_a_ × F_g_ and F_h_ were then compared to literature reported values. These predicted bioavailability numbers can serve as the fourth level of predictive similarity.

**Software and graphical user interface (GUI).** Validated components from previous ABMs were reused [7,9,24–27]. Our model was written in Java (Java 8) by using the MASON library, mason.19.jar, [28] (http://cs.gmu.edu/~eclab/projects/mason/), which is a fast discrete-event multi-agent simulation library. Models were developed, assembled and simulated within NetBeans IDE (https://netbeans.org/) and Eclipse (https://eclipse.org). The source code is provided in the Supporting Information. Output data files were processed, graphed, and analyzed via R (R3.1.1) (http://www.r-project.org/) within RStudio (http://www.rstudio.com/). The plyr package was used for data manipulation [29], the PK package for PK analyses [30], and the ggplot2 package for data visualization [31].

A graphical user interface was developed by using the MASON library, mason.19.jar, to better visualize every aspect of the *in silico* experiment. The GUI included a console for simulation start, pause and stop functions, an overview of the ISDAT, and a real-time chart for the amount of drug and metabolite in the systemic blood space. A GUI-enabled simulation video is available in the Supporting Information. Readers are strongly advised to view the video for better understanding of the model.

## Results

### Model structure and parameters

**Fig 2:**
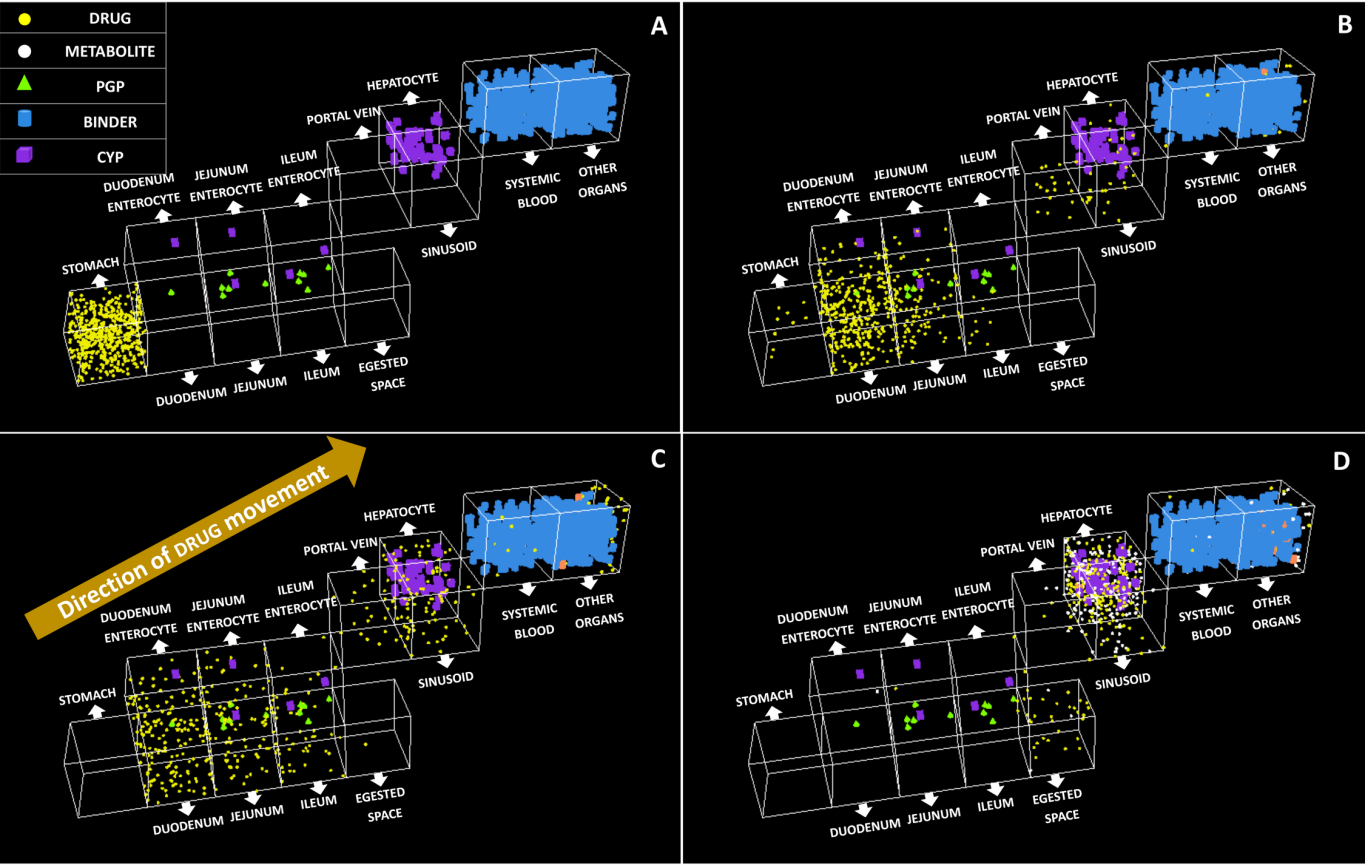
Snapshots of model during a single simulation run (using oral clonazepam administration as an example). (A) At the start of the simulation, drug objects (yellow dots) are placed in the stomach; cyps in enterocytes and hepatocyte, and binders in systemic blood and other organs. (B) Drugs moving across the enterocytes and towards systemic blood, simulating oral absorption. (C) At around C_max_, most drugs are present in the systemic blood, being bound to binders. (D) During elimination, drugs objects are being converted into metabolite (white dots), predominantly in the hepatocyte.

The model structure of ISDAT is depicted in Fig 1. Snapshots of ISDAT during a single simulation is presented in Fig 2. Model parameters of the system are presented in Table 1; they are held to be the same for both drugs. Drug-specific parameters are presented in Table 2. Experiment-related parameters are shown in Table 3.

**Table 1:** Final model parameters–system parameters.

Parameters	Remarks	space	Values
**systemSize**	Size of all spaces		20 × 20 × 20
**spacepH**	*In silico* pH of spaces	GI lumen	2.5–6.0 ^a^
		Cell and Blood	7.4
**flowRate**	Flow rate as biased movement in X direction in grid points per step	GI lumen	0.01–1.0 ^a^
		Blood	1.0–5.0 ^a^
**poresFrac**	Pores / tight junction on membrane as proportion of membrane area	GI lumen / Enterocytes	0.02
**flowParams**	Flow parameters: Depth of liquid flow between spaces as a proportion of space width	stomach to duodenum	0.5
		GI lumen	0.001–0.2 ^a^
		Blood	0.5–1.0 ^a^
	Flow parameters: Fraction of liquid flow between spaces as a proportion of flow area	stomach to duodenum	0.9
		GI lumen	0.125–0.2 ^a^
		Blood	0.3–1.0 ^a^
**TransitParams**	Transit parameters: fraction of space adjacent to membrane interface as proportion of space height	GI lumen / Enterocytes	0.5–0.8 ^a^
		Enterocytes / Portal Vein	0.8
		Hepatocyte / Sinusoid	0.1
	Transit parameters: membrane leakiness as a fraction of membrane area	GI lumen / Enterocytes	0.5–0.8 ^a^
		Enterocytes / Portal Vein	0.8
		Hepatocyte / Sinusoid	0.1
	Transit parameters: cellular uptake frequency as probability per step	GI lumen / Enterocytes	0.0
		Sinusoid / Hepatocyte	0.2
	Transit parameters: cellular uptake fraction as proportion of membrane area	GI lumen / Enterocyte	0.0
		Sinusoid / Hepatocyte	0.2
**Enzymes**	cyp expression levels (number of cyp objects)	Enterocytes	1, 2, 2
		Hepatocyte	60
	maxActiveSites (maximum metabolic capacity per cyp object per step)	cyp	1
	Neighborhood (number of grids in the neighborhood of the cyp)	cyp	9
**Transporters**	pgp expression levels (number of pgp objects)	Enterocytes	1, 5, 5
	maxActiveSites (maximum transport capacity per pgp object per step)	pgp	1
	Neighborhood (number of grids in the neighborhood of the pgp)	pgp	9
**Binders**	binder expression level (number of binder objects in systemic blood)	systemic blood	200
	maxActiveSites (maximum binding capacity per binder object in systemic blood)	binder	3
	Neighborhood (number of grids in the neighborhood of the binder in systemic blood)	binder	3
	binder expression level (number of binder objects in other organs)	other organs	500
	maxActiveSites (maximum binding capacity per binder object in other organs)	binder	1
	Neighborhood (number of grids in the neighborhood of the binder in other organs)	binder	1

^a^: The ranges include all the values of a vector of parameters and for each individual parameter, its value is fixed.

**Table 2:** Final model parameters–drug parameters.

Parameters	Remarks	Midazolam	Clonazepam
***MW***	*In silico* Molecular weight	325.7	315.7
***logP***	*In silico* Logarithm of partition coefficient	3.6	2.41
***Pka***	*In silico* acid/base equilibrium constant	6.1	1.86, 11.89
**pgpSubstrate**	Substrate of pgp	true	true
**AffinityPgp**	Affinity to pgp as probability of binding	0.1	0.1
**releaseProbPgp**	Release from pgp as probability of release	1.0	1.0
**cypSubstrate**	Substrate of cyp	True	True
**AffinityCYP**	Affinity to cyp as probability of binding	0.2	0.2
**releaseProbCyp**	Release from cyp as probability of release	1.0	1.0
**metabolizeProbCyp**	Metabolic rate as probability of metabolism	0.4	0.02
**binderSubstrate**	Substrate of binder	True	True
**AffinityBinder**	Affinity to binder as probability of binding	1.0	1.0
**releaseProbBinder**	Release from binder as probability of release	0.05	0.05

**Table 3:** Final model parameters–experiment parameters.

Parameters	Remarks	Midazolam	Clonazepam
**Steps**	Simulation experiment duration: counterpart for time	2500	10000
**TimeScale (h)**	Scaling factor between simulation steps and time	0.01	0.01
**numSolutes**	Number of drug objects: counterpart for dose administered	3274	500
**MeasureScale (ng/mL)**	Scaling factor between *in silico* amount and concentration	0.82	0.948

### Simulated profiles for midazolam and clonazepam

**Fig 3:**
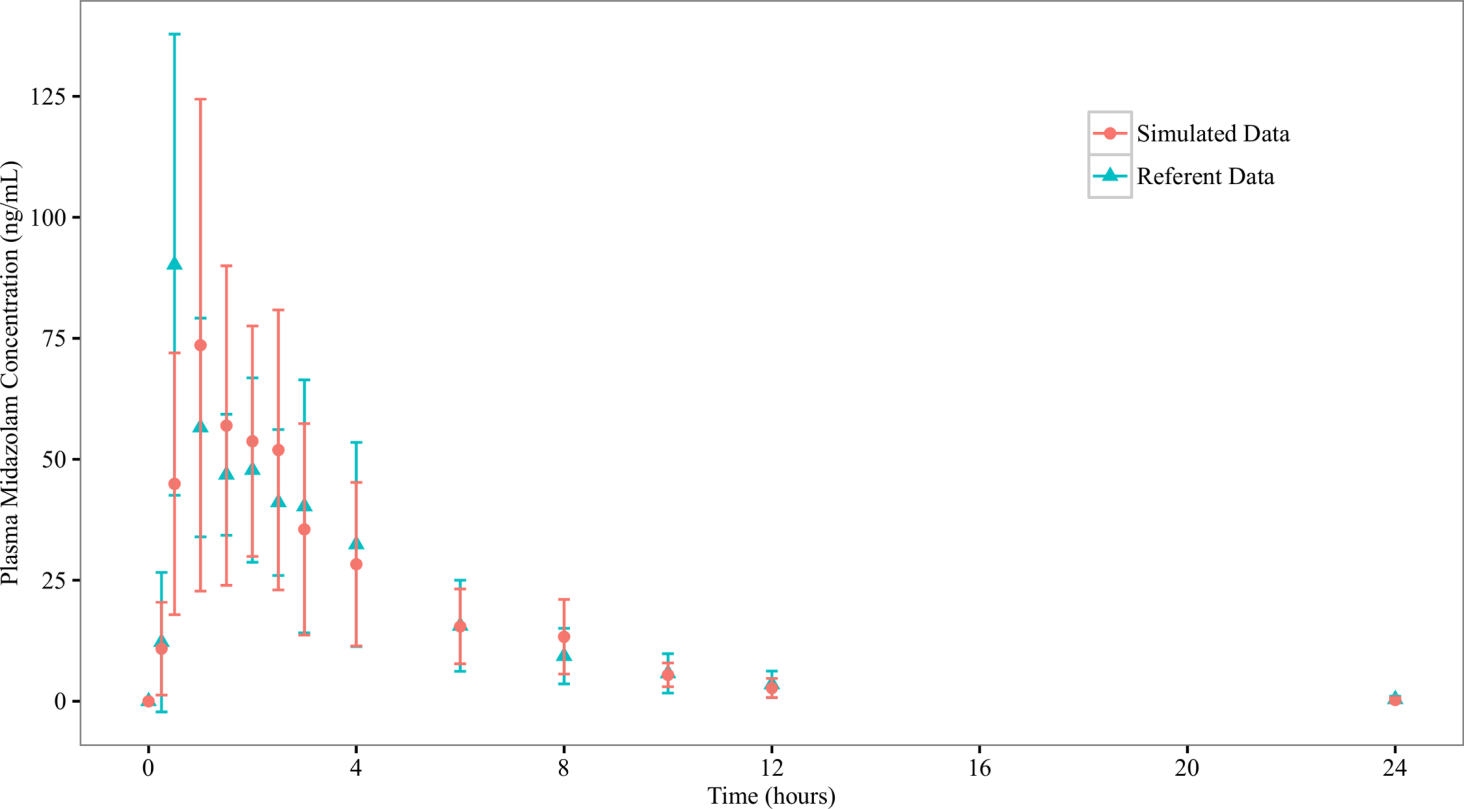
Referent and raw simulated profiles for midazolam. Graphed are mean (±1 SD) midazolam concentration profile from the referent clinical bioequivalence study, and the simulated mean midazolam concentration profile from the ISDAT.

**Fig 4:**
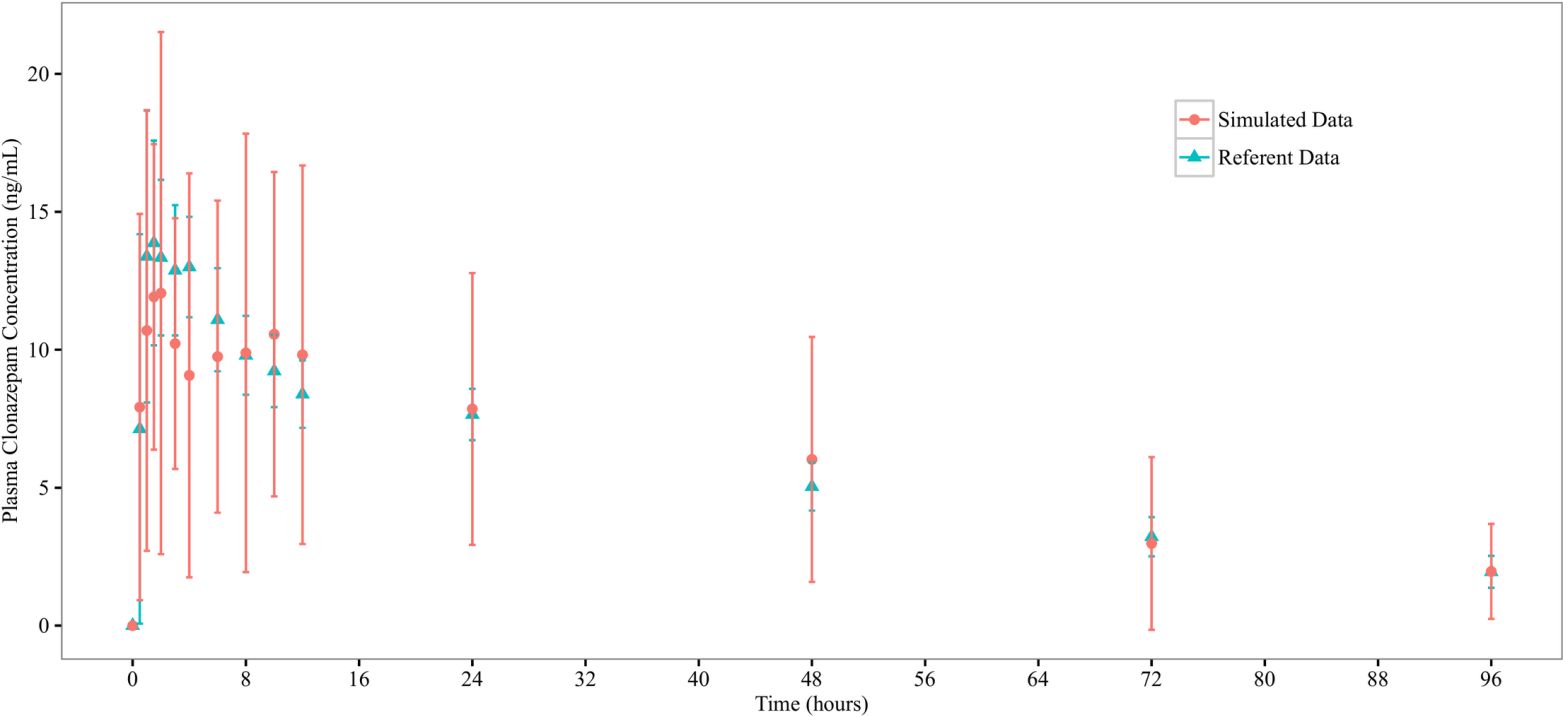
Referent and raw simulated profiles for clonazepam. Graphed are mean (±1 SD) clonazepam concentration profile from the referent clinical bioequivalence study, and the simulated mean clonazepam concentration profile from the ISDAT.

**Fig 5:**
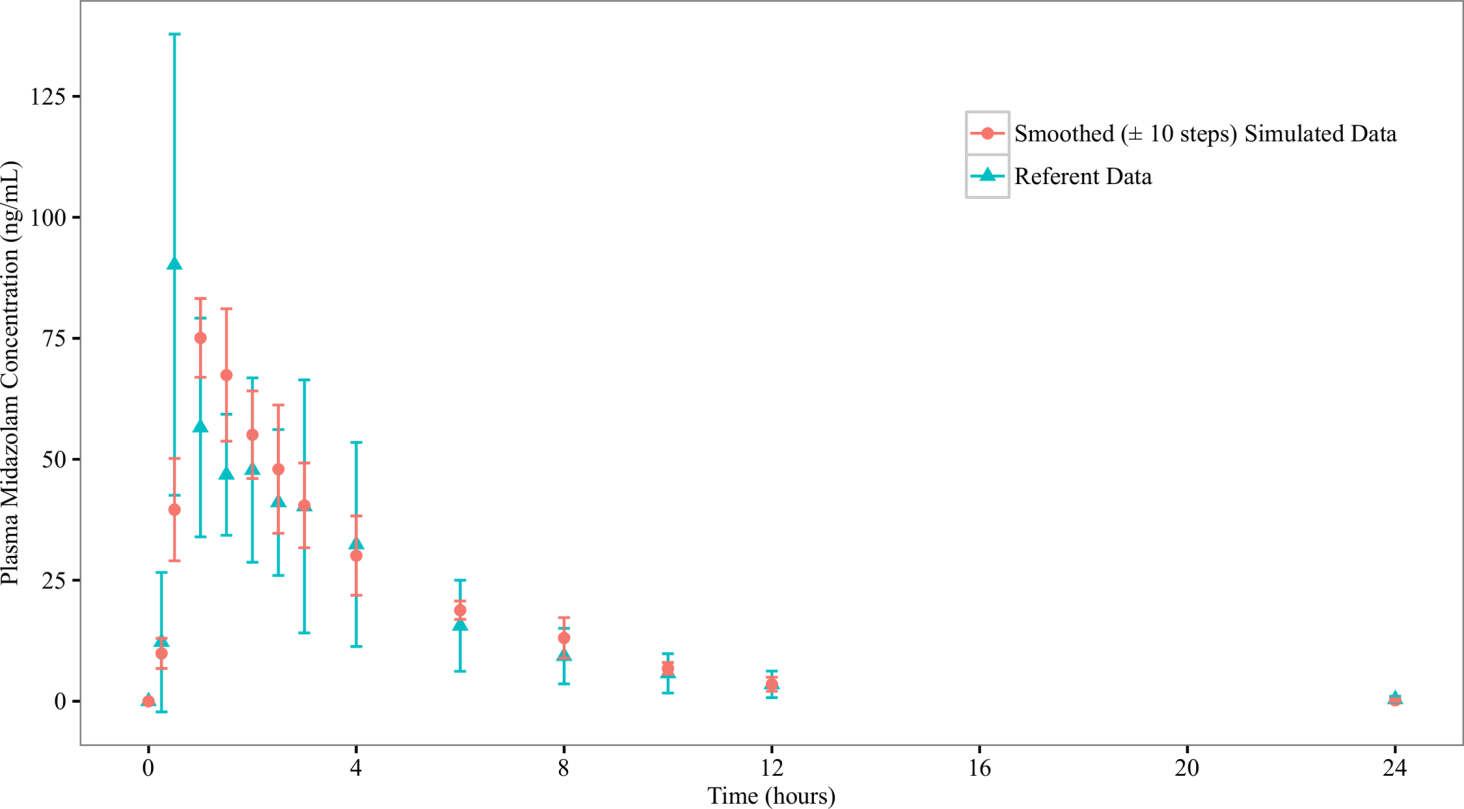
Referent and smoothed (±10 steps) simulated profiles for midazolam. Graphed are mean (±1 SD) midazolam concentration profile from the referent clinical bioequivalence study, and the smoothed (±10 steps) simulated mean midazolam concentration profile from the ISDAT.

**Fig 6:**
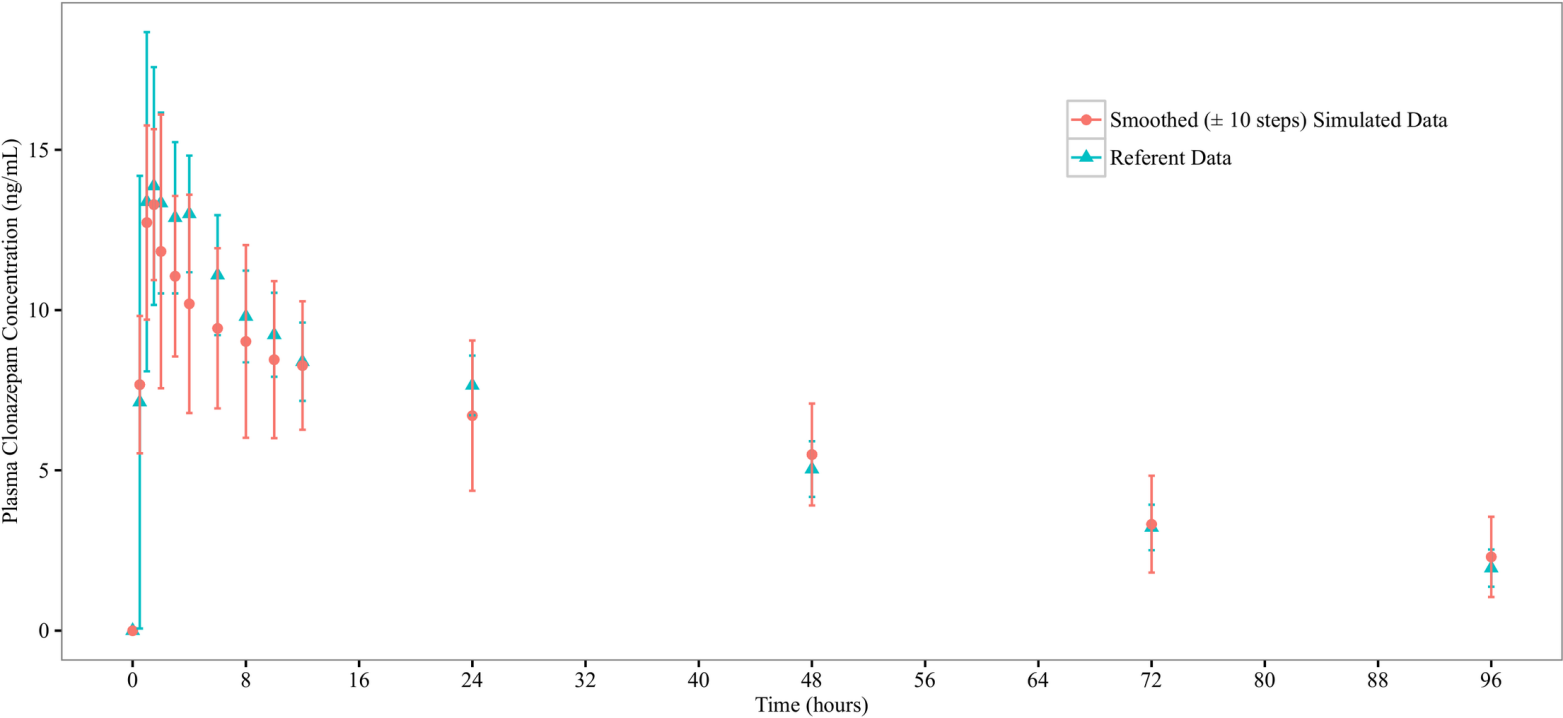
Referent and and smoothed (±10 steps) simulated profiles for clonazepam. Graphed are mean (±1 SD) clonazepam concentration profile from the referent clinical bioequivalence study, and the smoothed (±10 steps) simulated mean clonazepam concentration profile from the ISDAT.

ISDAT is aimed to simulate the mean concentration-time profiles from clinical studies. After a dose of compound was administered to the stomach space, measurements of the amount of compounds present in the systemic blood were made, with the sampling time points corresponding to those in the clinical studies. These serve as the raw simulation profiles (Figs 3 and 4), which have great step-to-step variations. Therefore, a smoothing (±10 steps) procedure is taken, which reduces SD around the mean concentration values (Figs 5 and 6).

### PK parameters and similarity criteria

PK parameters were calculated in both the clinical bioequivalence study and the *in silico* simulated experiment, which are presented in Table 4 using the smoothed (±10 steps) profiles. Key PK parameters are comparable between the simulated experiment and clinical study. Similarity criteria at the pre-specified levels are presented in Table 5.

**Table 4:** PK parameters (Mean ±1 SD) for midazolam and clonazepam.

PK Parameters	Midazolam (N = 15)		Clonazepam (N = 14)	
	Referent	Simulated ^b^	Referent	Simulated ^b^
**AUC_po (ng∙h∙mL^-1^)**	295.81 ± 125.50	318.83 ± 31.01	529.08 ± 80.79	523.71 ± 74.48
**C_max_ (ng∙mL^-1^)**	101.39 ± 33.04	77.81 ± 7.30	16.20 ± 4.28	15.96 ± 3.30
**T_max_ (h) ^**a**^**	0.5	1.00	1.50	1.50
**Kel (h^-1^)**	0.28 ± 0.07	0.28 ± 0.04	0.021 ± 0.004	0.018 ± 0.005
**T_1/2_ (h)**	3.74 ± 0.96	3.71 ± 0.66	49.02 ± 7.79	60.86 ± 18.12
**CL/F (L∙h^-1^)**	57.72 ± 19.31	47.47 ± 4.72	3.88 ± 0.68	3.89 ± 0.55
**V/F (L)**	205.20 ± 56.75	175.86 ± 35.60	186.84 ± 25.25	230.06 ± 46.81
**AUC_iv (ng∙h∙mL^-1^)**	-	687.72 ± 36.56	-	657.64 ± 93.20
**AUC_hpv (ng∙h∙mL^-1^)**	-	329.16 ± 24.62	-	574.73 ± 56.62

a: Median, instead of mean ±1 SD, is calculated for T_max_.

b: Simulated results being smoothed for ±10 steps.

**Table 5:** Similarity criteria for midazolam and clonazepam.

Similarity Criteria	Midazolam	Clonazepam
**1. Qualitative similarity—Visual match of data points in the concentration-time profiles**		
Simulated mean concentration within the mean ±1 SD range of referent values?	Met by all simulated data points.	Met by most simulated data points ^a^.
**2. Quantitative similarity**		
Weighted RMSE	0.51	0.92
Weighted MAPE	5.99%	8.43%
3. Descriptive similarity ^**b**^—Absolute difference (Relative difference)		
Difference between AUC	23.02 ng∙h∙mL^-1^ (7.8%)	-5.37 ng∙h∙mL^-1^ (1.0%)
Difference between C_max_	-23.58 ng∙mL^-1^ (23.3%)	-0.24 ng∙mL^-1^ (1.5%)
Difference between T_max_	0.5 h ^c^	0.0 h
Difference between Kel	0.0 h^-1^ (0.0%)	-0.003 h^-1^ (14.3%)
Difference between T_1/2_	-0.03 h (0.8%)	11.84 h (24.2%)
Difference between CL/F	-10.25 L∙h^-1^ (17.8%)	0.01 L∙h^-1^ (0.3%)
Difference between V/F	-29.34 L (14.3%)	43.22 L (23.1%)

a: There are 4 data points (27%) of the simulated mean profiles falling out of the mean ±1 SD range of the referent clinical profiles, namely, at 3, 10, 12, and 48 hour.

b: Differences between PK parameters are calculated with the referent clinical data as the baseline values. Simulated PK parameters are calculated based on the smoothing procedure (±10 steps).

c: Only absolute difference is shown for T_max_ because the time to reach C_max_ is quick after oral administration of midazolam and clonazepam yet highly variable based on the referent clinical data.

### Predicted bioavailability

**Fig 7:**
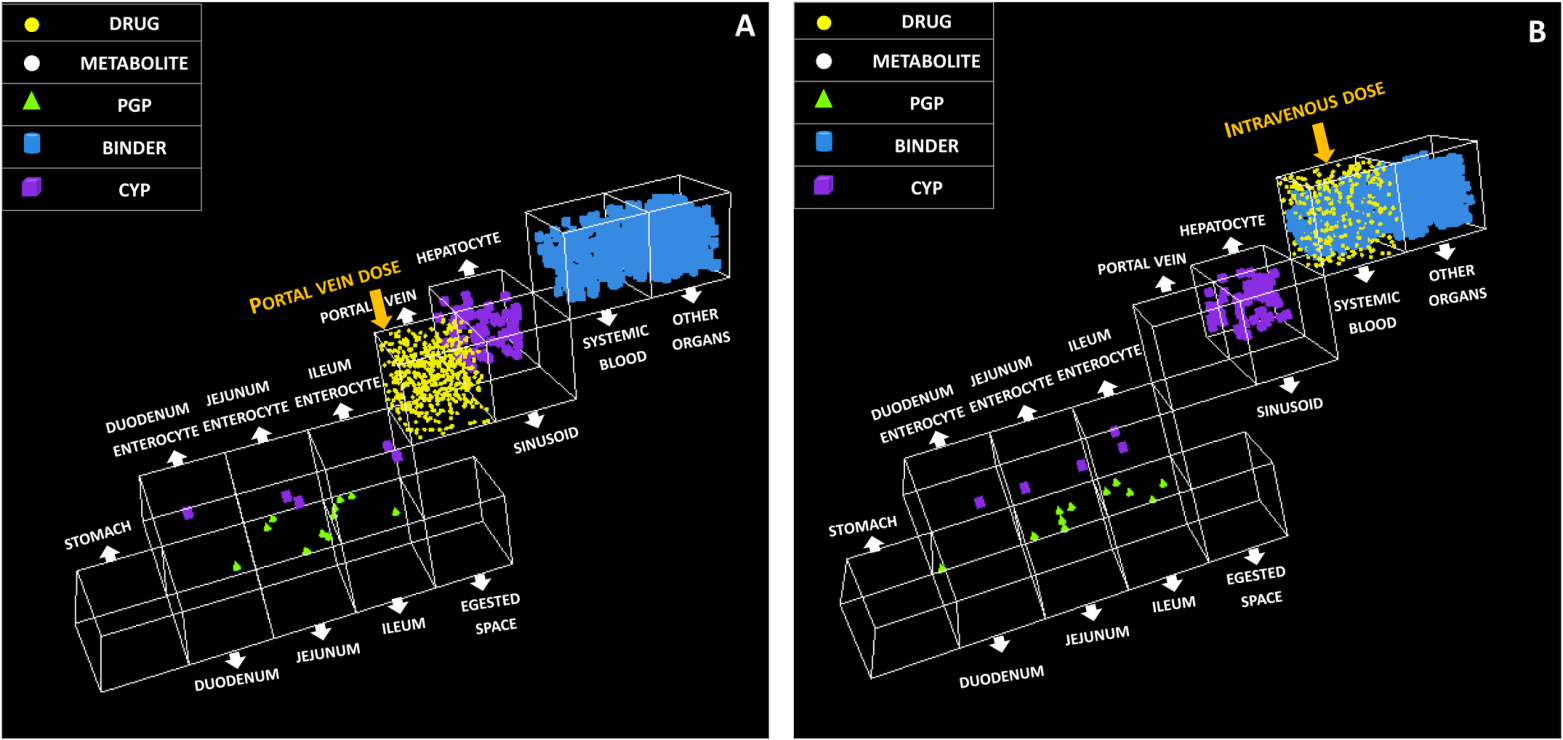
drug administration in the portal vein and systemic blood. (A) At the start of the simulation, drug objects (yellow dots) are placed in the portal vein; (B) At the start of the simulation, drug objects (yellow dots) are placed in the systemic blood.

Finally, the ability of the ISDAT to predict bioavailability, namely, F_a_ × F_g_, F_h_ and F was also assessed by dosing drug in portal vein and systemic blood (Fig 7).

However, since the bioavailability are not provided in the referent bioequivalence studies, values reported in literatures are referred to (Table 6). The predicted F_a_ × F_g_ was 93% (±30%) for midazolam (referent value: 48–76%) and 98% (±61%) for clonazepam (referent value: 93–95%) [32,33]; the predicted F_h_ was 48% (±7%) for midazolam (referent value: 36–57%) and 103% (±15%) for clonazepam (referent value: 95–97%) [33]; the predicted F was 44% (±11%) (referent value: 31–72%) for midazolam and 93% (±39%) (referent value: ≥ 90%) for clonazepam [34,35]. In all cases, there is overlap in the range between the literature reported values and the mean ±1 SD interval of the simulated data, therefore all predicted bioavailability values are acceptable.

**Table 6:** Predicted bioavailability for midazolam and clonazepam.

Bioavailability	Midazolam (N = 15)		Clonazepam (N = 14)	
	Referent ^a^	Simulated	Referent ^a^	Simulated
**F**_**a**_ **× F**_**g**_	48–76%	93% ± 30%	93–95%	98% ± 61%
**F**_**h**_	36–57%	48% ± 7%	95–97%	103% ± 33%
**F**	31–72%	44% ± 11%	>90%	93% ± 39%

a: Values reported in references [32–35].

## Discussion

### Prototype for *in silico* drug absorption simulation

In this project, a prototypical device, ISDAT, is built to simulate oral drug absorption in human. The device incorporates important multi-level physicochemical, physiological and PK processes, namely, passive permeation, intestinal motility, blood circulation, intestinal metabolism, and hepatic extraction, which is then validated against the clinical data of midazolam and clonazepam. ISDAT can produce systemic phenomena acceptably similar to the clinical data, as measured by mean concentrations, PK parameters, as well as by pre-specified similarity criteria. In sum, ISDAT stands as a computational, yet biomimetic, analogue of human GI tract for simulation of oral drug absorption.

### Two drugs, one system

Importantly, although the drug-specific parameters reflected the properties of two test drugs, the system parameters held the same for both. Also, the range of concentration and time for the two drugs were very different: 24 hours with a C_max_ of 90.21 ng/mL for midazolam, and 96 hours with a C_max_ of 13.48 ng/mL for clonazepam. Of note, although the number of metabolic enzymes in ISDAT was the same in experiments for both drugs, the calculated clearance values were 47.47 ± 4.72 L/h and 3.89 ± 0.68 L/h for midazolam and clonazepam (simulation data smoothed ± 10 steps), respectively. The difference in clearance can be explained by different affinities and access to the enzymes, and hence different metabolic rates. Therefore, it can be concluded that this model is not only adequate for a single drug, but also for two markedly different drugs, over different time horizons, and may also be useful for other drugs as well.

### Parameter search and values

The model developed here has three sets of parameters: system parameters related to the modeling of GI physiology; drug parameters related to the properties of administered drugs; and experiment parameters related to the clinical bioequivalence study. For the experiment parameters, only the *TimeScale* and the *MeasureScale* parameters were chosen with consideration of achieving similarity; others were chosen to simulate the referent experiments, and therefore all of them were fixed throughout the model’s development. The choice of the system and drug parameters values are mostly based on *a priori* knowledge and assumption about the drugs and GI physiology. Because relational grounding was adopted [8], it is the relative magnitude of the parameters that mattered. For example, as represented by the transit parameters of the gi tract, jejunum, when compared to duodenum, has higher effective absorption area. Therefore, the absorptive process is expected to be more extensive in the jejunum, which reflects the knowledge about drug absorption and GI physiology. However, we did not attempt to map the model parameters, by one-to-one correspondence, to the actual physical flow rates and surface areas of the human GI tract; this is because such mapping would require absolute grounding, which would in turn limit the further development of future iterations of the model [8]. As such, we are cautious about directly interpreting the model parameters in the absolute sense, or about making predictions or comparisons based on their absolute values; rather, our focus is on the overall assembly of the components to form a consistent ISDAT that can mimic oral drug absorption of two drugs.

### Validation

ISDAT was validated to different degrees. To start with, each of the components was verified individually to ensure that they functioned as designed. In terms of face validation, the assembly, as a whole, was inspired by our knowledge about human anatomy and physiology, as well as by the general principles of drug absorption to ensure that the components were working as intended.

Then, meeting the similarity criteria in the specified three levels, qualitative-quantitative-descriptive stands as the functional validation. All of them were within 25% of the corresponding mean referent values from the clinical data, except for T_max_, which is inherently highly variable. Hence, on top of the similarity of sampled time points between the clinical data and the simulated results, the PK parameters, as summary indicators for the whole concentration-time profile, were similar as well.

Last but not least, predicated bioavailability values, F_a_ × F_g_, F_h_ and F, being similar to literature reported ones gave yet another degree of predictive validation. With these, we argue that although our model may not be a one-to-one mapping to actual human gastrointestinal physiology, it is nevertheless consistent with, and complementary to, current models about pharmacokinetics of oral drug absorption.

We recognize that more rigorous validation strategies are possible, for example, with dividing the data into training and validation datasets, with a third drug, or with more stringent similarity criteria. However, each of the above strategies has its own limitation. For instance, matching a smaller subset of observations (as required in dividing the data into training and validation sets) is much easier than matching the whole available data. Using a third drug could introduce bias if that third drug has vastly different PK properties, say, it being a substrate of additional enzymes or transporters. Meeting more stringent similarity criteria could mean underrepresenting the interindividual variability presented in the clinical data.

In all, we think that the current validation is adequate for the prototypical device.

### Limitations

There are a few limitations in our model, though. Firstly, the drugs were implemented as fully soluble, and so there was no solubility limit in the simulation. However, in both the referent clinical data and simulated data, both midazolam and clonazepam exhibited rapid absorption, and the solubility did not appear to limit the drug absorption extent. The possible solubility limit in blood for clonazepam was simulated with extensive protein binding. Hence, we argue that, for the two test drugs, the solubility limit was not a concern. In the future research, the implementation of a solubility limit for poorly soluble drugs will need to be a priority. Secondly, wide stochastic variability between simulations was shown in our simulations. This is a limitation of agent-based simulations, when compared to simulations based on systems of differential equations. It was not uncommon to see simulated results varied by > 15% from run to run, comparable to previous reports [7,9,36]. This degree of variability is consistent with our other projects involving agent-based simulations, as well as with other researchers’ experiments. Stochasticity, inherent in ABM, can be classified as run-to-run stochasticity and step-to-step stochasticity. Therefore, we chose to repeat our simulations by 15 or 14 times mapping to 15 or 14 test subjects to ensure that the overall mean values were reliable considering run-to-run stochastic effect. In addition, we adopted the smoothing procedure to minimize step-to-step variability while maintaining the precision of measurements. Currently simulated data are smoothed at 10 steps, which traverses 20 steps in total and maps to 0.2 hour, less than the minimum sampling interval (0.25 hour) in the referent studies.

Still, we did not represent the observed interindividual variability from the clinical data. Although we demonstrated the ability to simulate interindividual variability in a different study, by setting different system parameters for each individual, in this study we simulated only to match the mean values of referent data because our primary focus in the current study was to show that the model was capable of simulating two drugs even if the system parameters were the same. A logical next step would be to simulate each of the individual concentration-time profiles with slightly different systems parameters, so that we can better simulate the observed inter-individual variability.

### Potential applicability

#### Altered physiology, time-variant systems, what-if scenarios

Because of the inherent variability in the ABM as well as the clinical data, we do not expect that our model can give quantitatively accurate predictions at this stage. Also, we did not attempt to use ISDAT as an analysis tool to replace the conventional PBPK or noncompartmental analysis for bioequivalence. Rather, we argue that the model is more useful for *in vitro–in vivo* translation, or for qualitative exploration of what-if scenarios, and to provide concrete actionable answers to what-if questions that arise during the research and development of orally administered therapeutics. There are scenarios that are difficult to test in the clinical setting, yet they could be clinically relevant in altering drug absorption and disposition. For example, what if the subject has a GI motility disease where the contents of the GI tract move slower than normal? The flow parameters can be decreased. What if the subject is an extensive metabolizer? The enzyme’s metabolic parameters can be changed. What if hepatic enzyme amount is reduced in liver injury or intestinal enzyme amount happens to be higher in some individuals? The enzyme’s expression levels can be altered. For all these above examples, the ISDAT can offer a qualitative prediction, by simulating with simple changes in a few model parameters. We present these in Supporting Information (S1–S6 Figs). More importantly, because of the stochastic nature of the ABM, the expected variation could be simulated naturally as well. In contrast, simulation of the above with a set of developed, continuous differential equation-based models may prove highly complicated, if not intractable. Therefore, ISDAT can be further developed to serve as an informative virtual laboratory, complementary to common PK modeling and simulation strategies, in helping to answer what-if questions.

#### Knowledge integration

Further-developed ISDAT is also expected to provide concrete instances of mechanisms for us to assemble, test, and verify or reject, mechanism-based hypotheses about the determinants of bioavailability [7]. By continuously and iteratively updating these mechanism-based analogues, we incrementally assemble better and better mechanism-based hypotheses–we accumulate current knowledge (and ignorance) about drug absorption, and have them represented in computational analogues with biomimetic algorithms [6,9,37]. The model allows us to gain deeper insights into the relevant, causal, mechanism-based details underlying and accounting for the unique, individual-specific PKs and bioavailability of different drugs and formulations. Successfully validated models can represent currently best theory for multiple aspects of system function instantiated. Knowledge, as well as uncertainty, could be progressively represented and integrated. Thus, resulting models become shared, interactive and observable pharmacology knowledge embodiments. These knowledge embodiments will complement the existing equation-based modeling and simulation methods and wet-lab models. When the current best theory fails to validate, we would have identified important gaps in the current theory. Together, they will be one further step in the shift of pharmaceutical research towards a rational, learn-and-confirm paradigm [38] and model-based drug development approach [39].

### Overall significance

In summary, we have developed a prototypical ISDAT, an *in silico* device which is capable of modeling oral drug absorption in human. The model can generate concentration-time profiles and PK parameters with an acceptable similarity to the clinical data. We believe that modeling and simulations with ISDAT will provide insights that cannot be achieved by *in vitro*, animal, and human experiments alone. In the long run, models like ISDAT can represent and integrate our knowledge about mechanisms about drug absorption into a dynamic executable platform.

## Conclusions

ISDAT, an agent-based model describing human oral drug absorption, was successfully developed to simulate clinical data of midazolam and clonazepam with acceptable similarity. Being a model complementary to the conventional equations, ISDAT is expected to serve as an invaluable platform in further research into the mechanisms of oral drug absorption.

## Supporting information

S1 Video A video for GUI demonstration of ISDAT. (MP4)

S1 Fig Smoothed (±10 steps) simulated profiles for midazolam in case of retarded (to 1%) stomach flow. Graphed are mean (±1 SD) concentration profile from the baseline simulated study (green triangles), and the smoothed (±10 steps) simulated results in the speculated scenario (red circles): retarded stomach flow.
(TIF)

S2 Fig Smoothed (±10 steps) simulated profiles for clonazepam in case of retarded (to 1%) stomach flow. Graphed are mean (±1 SD) concentration profile from the baseline simulated study (green triangles), and the smoothed (±10 steps) simulated results in the speculated scenario (red circles): retarded stomach flow.
(TIF)

S3 Fig Smoothed (±10 steps) simulated profiles for midazolam in case of enhanced (to 200%) cyp activity. Graphed are mean (±1 SD) concentration profile from the baseline simulated study (green triangles), and the smoothed (±10 steps) simulated results in the speculated scenario (red circles): enhanced cyp activity.
(TIF)

S4 Fig Smoothed (±10 steps) simulated profiles for clonazepam in case of enhanced (to 200%) cyp activity. Graphed are mean (±1 SD) concentration profile from the baseline simulated study (green triangles), and the smoothed (±10 steps) simulated results in the speculated scenario (red circles): enhanced cyp activity.
(TIF)

S5 Fig Smoothed (±10 steps) simulated profiles for midazolam in case of reduced (to 50%) hepatic cyp amount. Graphed are mean (±1 SD) concentration profile from the baseline simulated study (green triangles), and the smoothed (±10 steps) simulated results in the speculated scenario (red circles): reduced hepatic cyp amount.
(TIF)

S6 Fig Smoothed (±10 steps) simulated profiles for clonazepam in case of reduced (to 50%) hepatic cyp amount. Graphed are mean (±1 SD) concentration profile from the baseline simulated study (green triangles), and the smoothed (±10 steps) simulated results in the speculated scenario (red circles): reduced hepatic cyp amount.
(TIF)

S1 Table Concentration-time profiles of midazolam. (DOCX)

S2 Table Concentration-time profiles of clonazepam. (DOCX)

S3 Table PK parameters of midazolam. (DOCX)

S4 Table PK parameters of clonazepam. (DOCX)

S5 Table PK parameters of midazolam at speculated scenarios. (DOCX)

S6 Table PK parameters of clonazepam at speculated scenarios. (DOCX)

S1 File Raw referent clinical data. (XLS)

S2 File Simulation data. (RAR)

S3 File Source code of ISDAT. (RAR)
