# Supplementary figures and images for: In silico drug absorption tract: An agent-based biomimetic model for human oral drug absorption
Source: PLoS One. 2018 Aug 31;13(8):e0203361. doi: 10.1371/journal.pone.0203361 (PMC6118387; doi:10.1371/journal.pone.0203361)

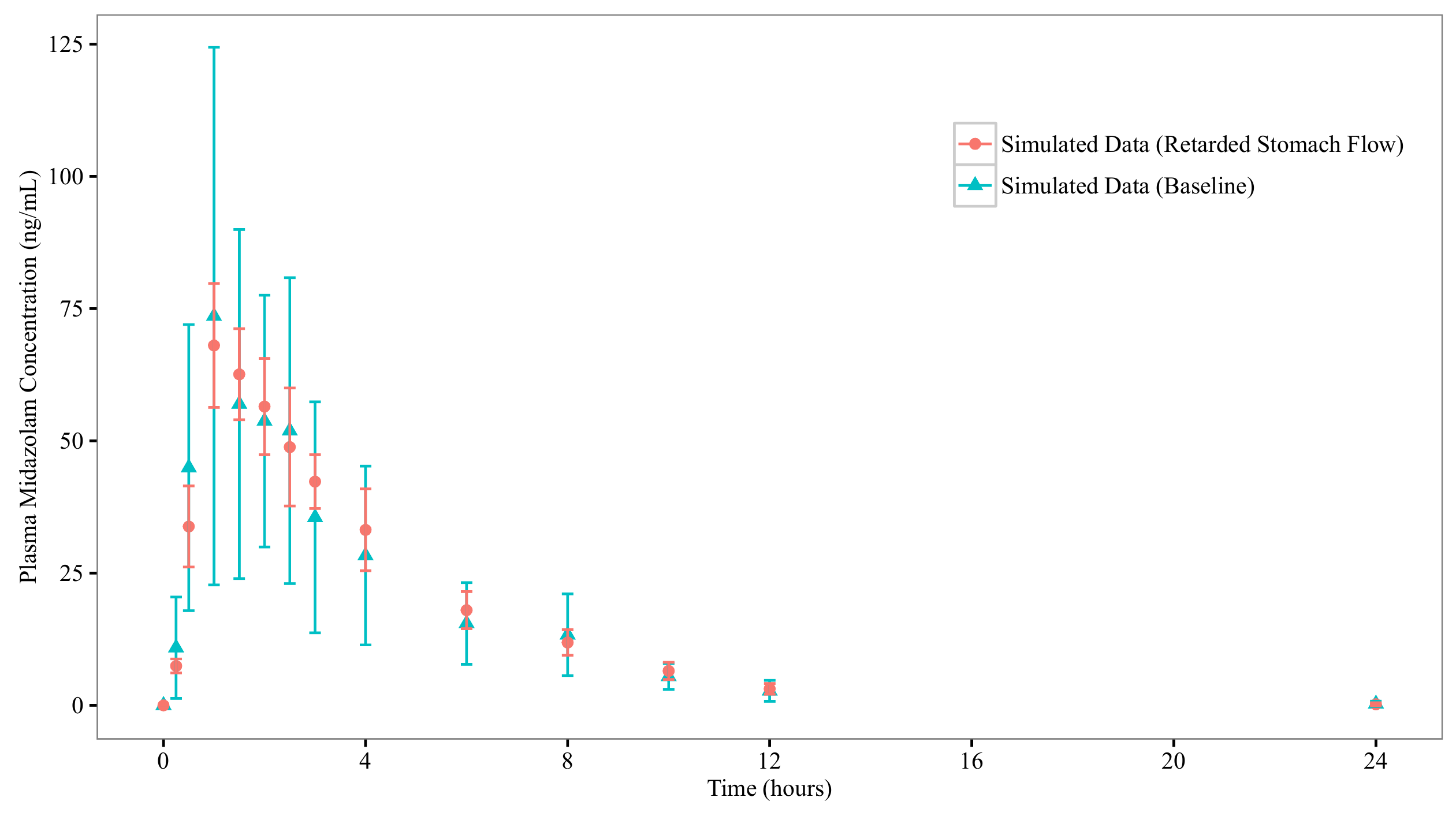

Supplement: S1 Fig — Graphed are mean (±1 SD) concentration profile from the baseline simulated study (green triangles), and the smoothed (±10 steps) simulated results in the speculated scenario (red circles): retarded stomach flow. (TIF) [file pone.0203361.s002.tif]

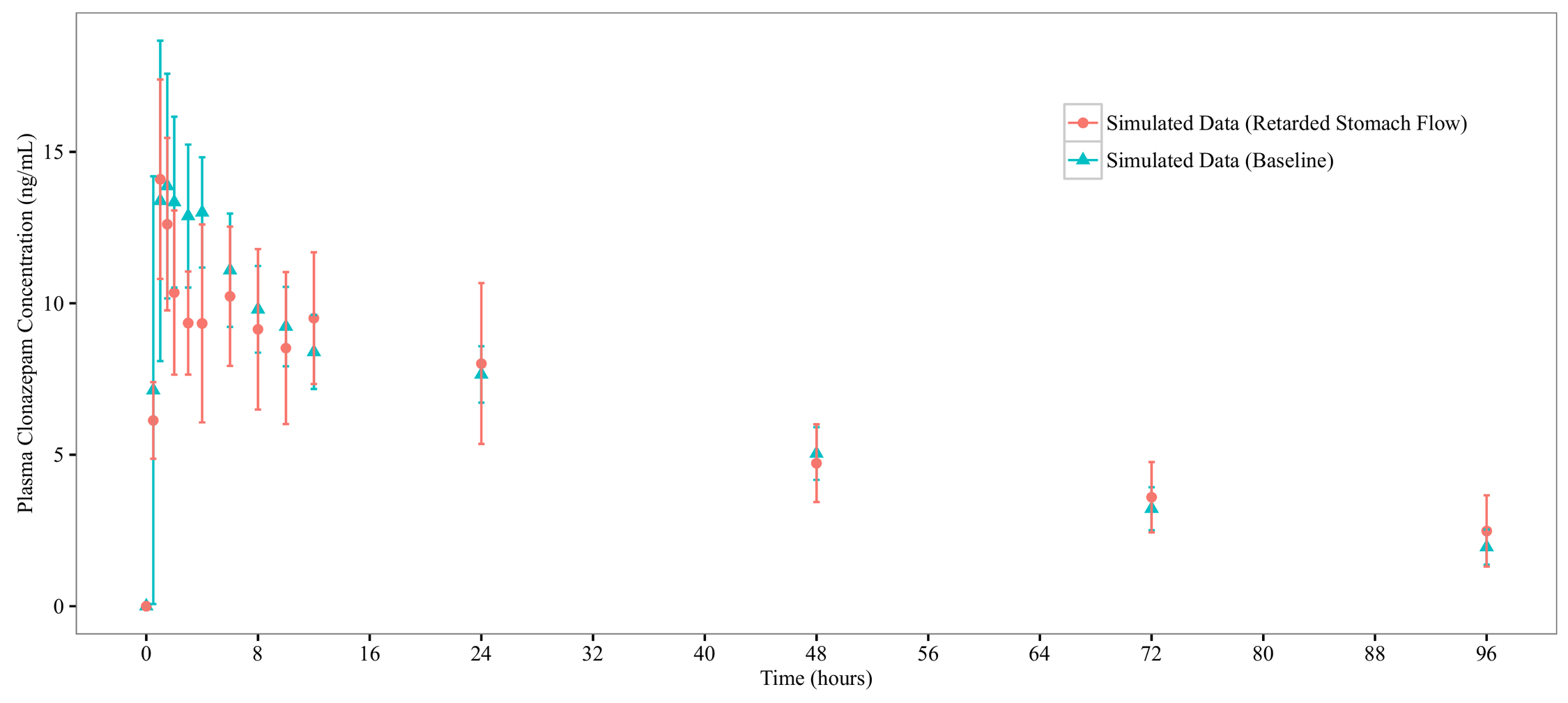

Supplement: S2 Fig — Graphed are mean (±1 SD) concentration profile from the baseline simulated study (green triangles), and the smoothed (±10 steps) simulated results in the speculated scenario (red circles): retarded stomach flow. (TIF) [file pone.0203361.s003.tif]

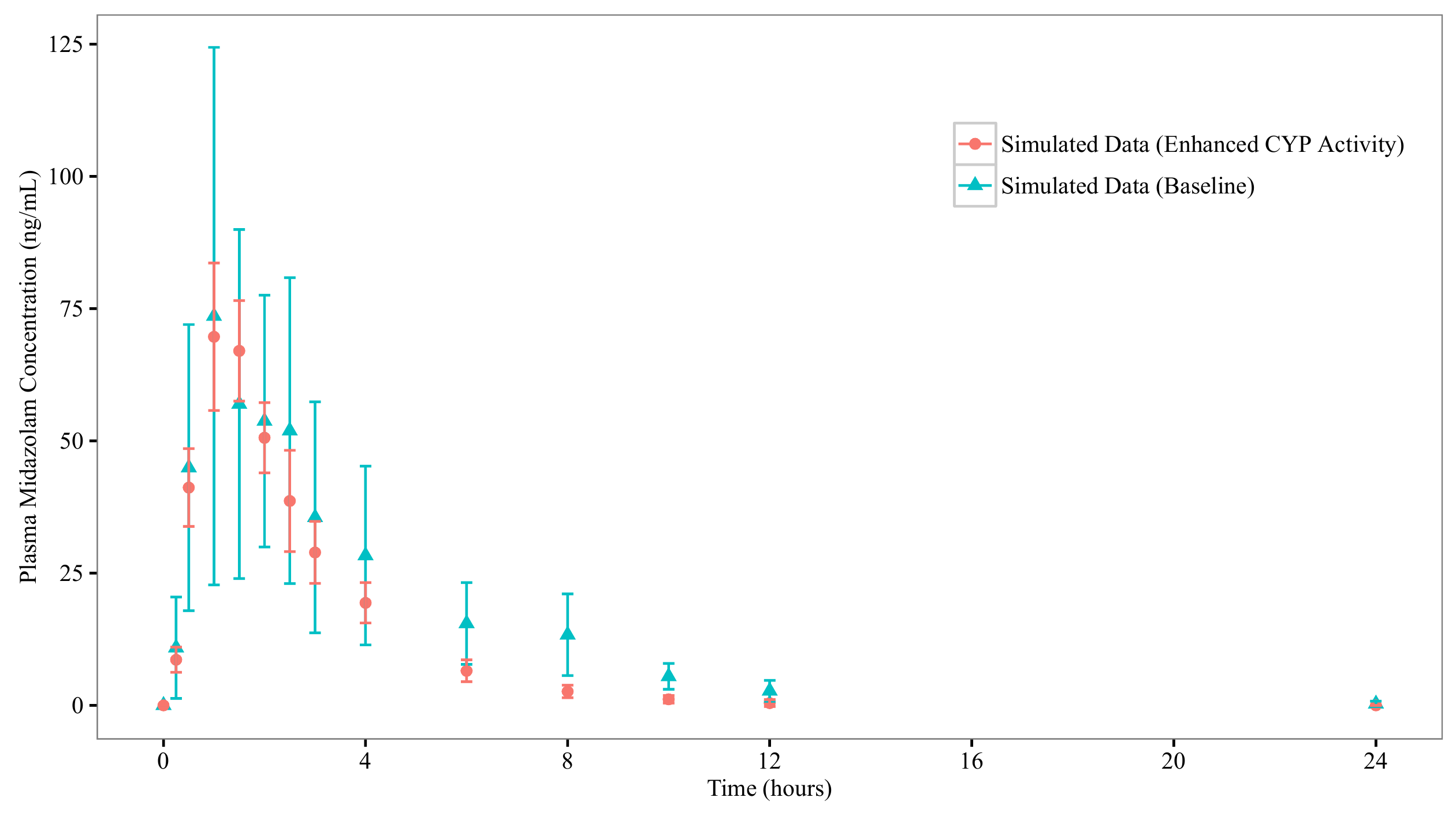

Supplement: S3 Fig — Graphed are mean (±1 SD) concentration profile from the baseline simulated study (green triangles), and the smoothed (±10 steps) simulated results in the speculated scenario (red circles): enhanced cyp activity. (TIF) [file pone.0203361.s004.tif]

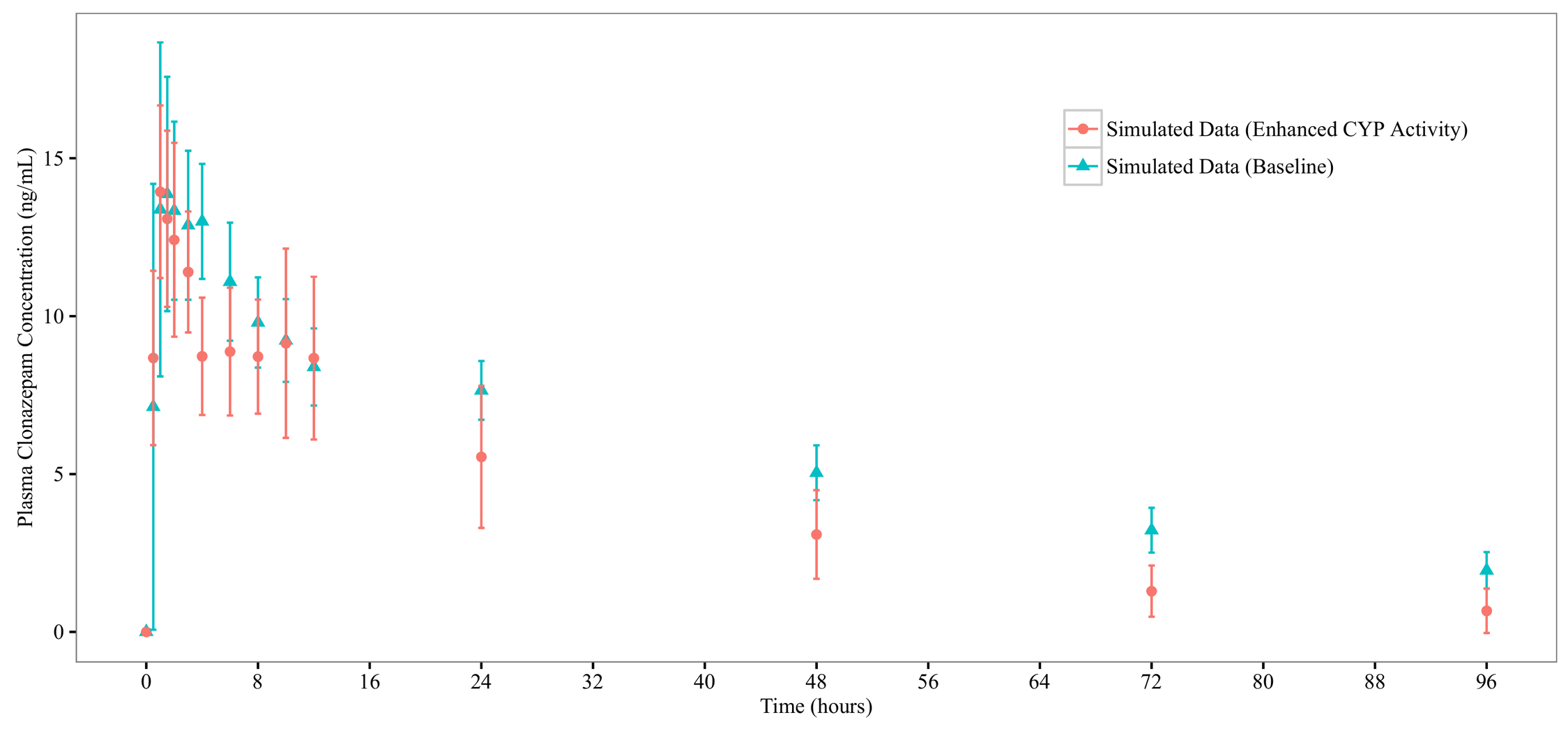

Supplement: S4 Fig — Graphed are mean (±1 SD) concentration profile from the baseline simulated study (green triangles), and the smoothed (±10 steps) simulated results in the speculated scenario (red circles): enhanced cyp activity. (TIF) [file pone.0203361.s005.tif]

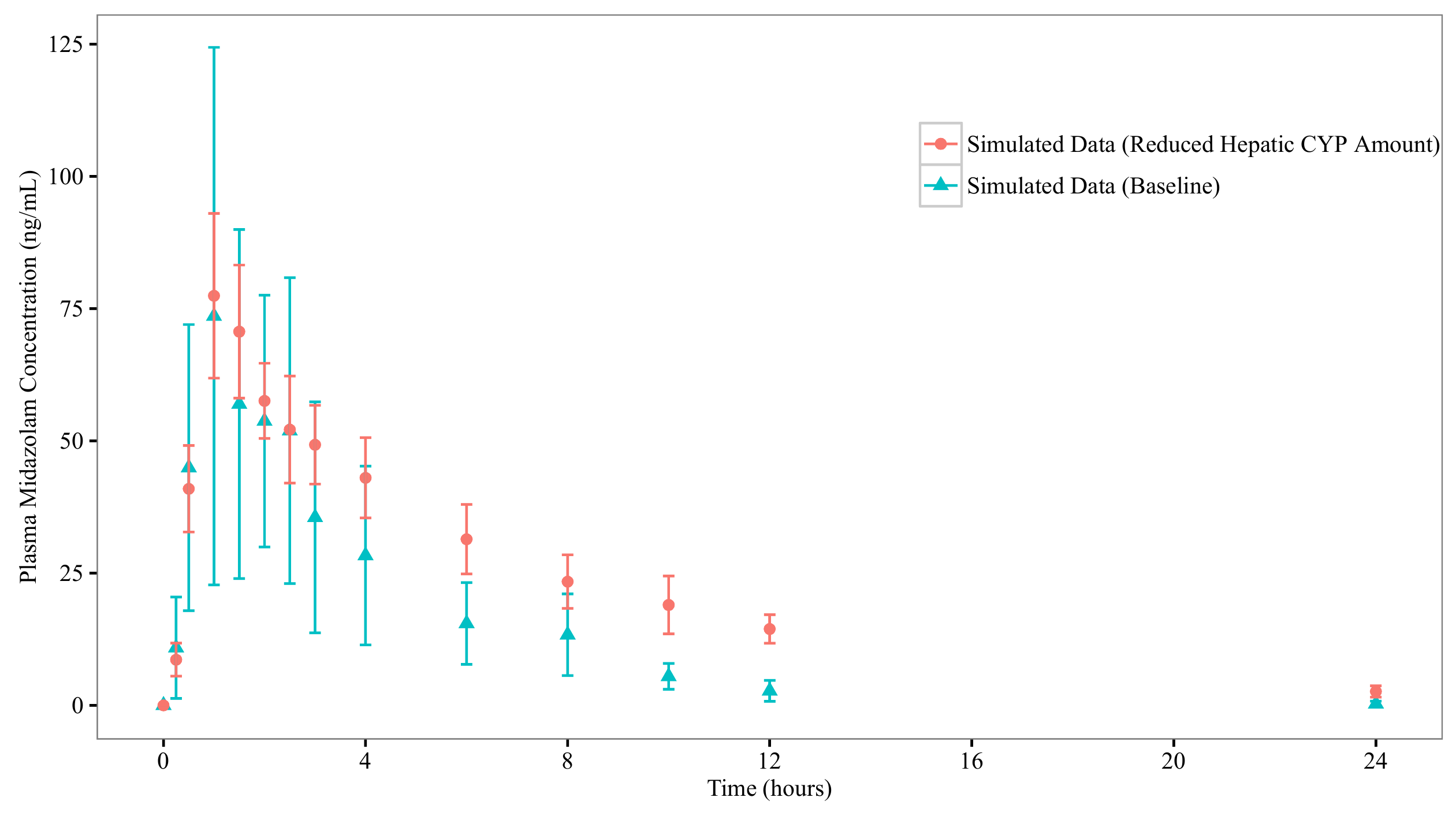

Supplement: S5 Fig — Graphed are mean (±1 SD) concentration profile from the baseline simulated study (green triangles), and the smoothed (±10 steps) simulated results in the speculated scenario (red circles): reduced hepatic cyp amount. (TIF) [file pone.0203361.s006.tif]

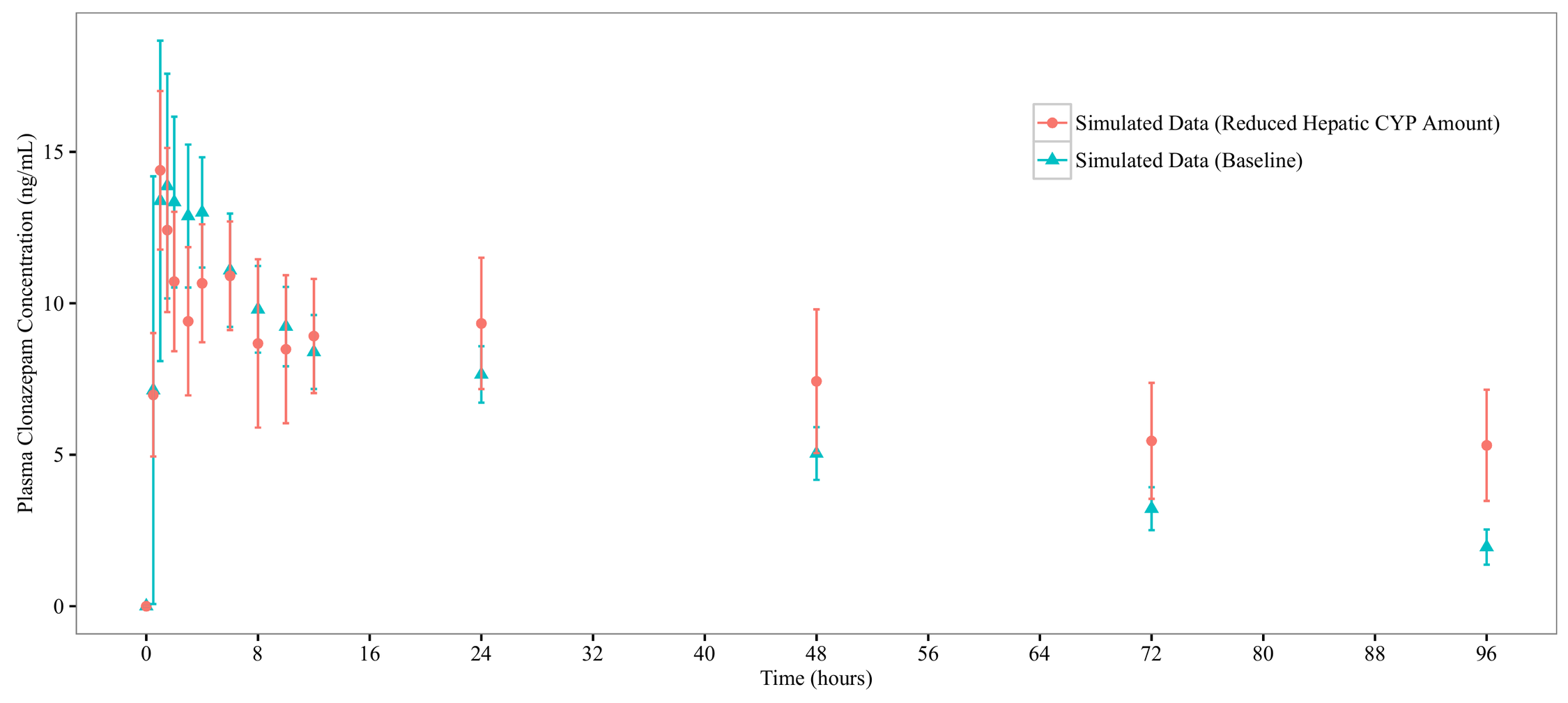

Supplement: S6 Fig — Graphed are mean (±1 SD) concentration profile from the baseline simulated study (green triangles), and the smoothed (±10 steps) simulated results in the speculated scenario (red circles): reduced hepatic cyp amount. (TIF) [file pone.0203361.s007.tif]
