# Supplementary material for: In silico drug absorption tract: An agent-based biomimetic model for human oral drug absorption
Source: PLoS One. 2018 Aug 31;13(8):e0203361. doi: 10.1371/journal.pone.0203361 (PMC6118387; doi:10.1371/journal.pone.0203361)
Supplement: S1 Table — (DOCX) [file pone.0203361.s008.docx]

S1 Table. Concentration-time profiles (Mean ±1 SD) of midazolam (N=15)

|  | Concentrations (ng/mL) | | |
| --- | --- | --- | --- |
| Time (hours) | **Referent** | **Raw simulated** | **Smoothed ( ± 10 steps) simulated** |
| 0 | 0.00±0.00 | 0.00 ± 0.00 | 0.00 ± 0.00 |
| 0.25 | 12.21±14.41 | 10.88 ± 9.58 | 9.90 ± 3.12 |
| 0.5 | 90.21±47.64 | 44.94 ± 27.05 | 39.59 ± 10.58 |
| 1 | 56.56±22.60 | 73.58 ± 50.82 | 75.08 ± 8.14 |
| 1.5 | 46.81±12.51 | 56.96 ± 33.00 | 67.41 ± 13.69 |
| 2 | 47.77±19.06 | 53.74 ± 23.80 | 55.08 ± 9.04 |
| 2.5 | 41.08±15.08 | 51.93 ± 28.91 | 47.96 ± 13.26 |
| 3 | 40.26±26.14 | 35.53 ± 21.84 | 40.48 ± 8.75 |
| 4 | 32.41±21.08 | 28.32 ± 16.90 | 30.10 ± 8.18 |
| 6 | 15.6±9.40 | 15.47 ± 7.73 | 18.82 ± 1.90 |
| 8 | 9.33±5.73 | 13.34 ± 7.72 | 13.09 ± 4.18 |
| 10 | 5.75±4.06 | 5.47 ± 2.45 | 6.80 ± 1.21 |
| 12 | 3.48±2.75 | 2.73 ± 1.98 | 3.51 ± 1.46 |
| 24 | 0.41±0.63 | 0.27 ± 0.51 | 0.20 ± 0.28 |
