# Supplementary material for: In silico drug absorption tract: An agent-based biomimetic model for human oral drug absorption
Source: PLoS One. 2018 Aug 31;13(8):e0203361. doi: 10.1371/journal.pone.0203361 (PMC6118387; doi:10.1371/journal.pone.0203361)
Supplement: S2 Table — (DOCX) [file pone.0203361.s009.docx]

S2 Table. Concentration-time profiles (Mean ±1 SD) of clonazepam (N=14)

|  | Concentrations (ng/mL) | | |
| --- | --- | --- | --- |
| Time (hours) | **Referent** | **Raw simulated** | **Smoothed ( ± 10 steps) simulated** |
| 0 | 0.00 ± 0.00 | 0.00 ± 0.00 | 0.00 ± 0.00 |
| 0.5 | 7.13 ± 7.06 | 7.92 ± 7.00 | 7.67 ± 2.14 |
| 1 | 13.38 ± 5.29 | 10.70 ± 7.99 | 12.73 ± 3.03 |
| 1.5 | 13.87 ± 3.71 | 11.92 ± 5.54 | 13.29 ± 2.35 |
| 2 | 13.34 ± 2.82 | 12.05 ± 9.46 | 11.83 ± 4.27 |
| 3 | 12.88 ± 2.36 | 10.22 ± 4.54 | 11.06 ± 2.50 |
| 4 | 13.00 ± 1.82 | 9.07 ± 7.32 | 10.20 ± 3.40 |
| 6 | 11.09 ± 1.87 | 9.75 ± 5.66 | 9.43 ± 2.50 |
| 8 | 9.80 ± 1.43 | 9.89 ± 7.95 | 9.02 ± 3.01 |
| 10 | 9.23 ± 1.31 | 10.56 ± 5.88 | 8.45 ± 2.50 |
| 12 | 8.39 ± 1.22 | 9.82 ± 6.86 | 8.27 ± 2.00 |
| 24 | 7.65 ± 0.93 | 7.85 ± 4.92 | 6.71 ± 2.34 |
| 48 | 5.04 ± 0.87 | 6.03 ± 4.44 | 5.49 ± 1.59 |
| 72 | 3.22 ± 0.71 | 2.98 ± 3.13 | 3.32 ± 1.51 |
| 96 | 1.95 ± 0.58 | 1.96 ± 1.72 | 2.30 ± 1.25 |
