# Supplementary material for: In silico drug absorption tract: An agent-based biomimetic model for human oral drug absorption
Source: PLoS One. 2018 Aug 31;13(8):e0203361. doi: 10.1371/journal.pone.0203361 (PMC6118387; doi:10.1371/journal.pone.0203361)
Supplement: S3 Table — (DOCX) [file pone.0203361.s010.docx]

S3 Table. PK parameters (Mean ±1 SD) of midazolam (N=15)

| PK Parameters | Referent | Raw simulated | Smoothed ( ± 10 steps) simulated |
| --- | --- | --- | --- |
| AUC_po (ng∙h∙mL^-1^) | 295.81 ± 125.50 | 296.13 ± 65.75 | 318.83 ± 31.01 |
| C_max_ (ng∙mL^-1^) | 101.39 ± 33.04 | 104.19 ± 34.95 | 77.81 ± 7.30 |
| T_max_ (h) ^a^ | 0.50 | 1.50 | 1.00 |
| Kel (h^-1^) | 0.28 ± 0.07 | 0.28 ± 0.07 | 0.28 ± 0.04 |
| T_1/2_ (h) | 3.74 ± 0.96 | 3.86 ± 1.02 | 3.71 ± 0.66 |
| CL/F (L∙h^-1^) | 57.72 ± 19.31 | 53.23 ± 12.98 | 47.47 ± 4.72 |
| V/F (L) | 205.20 ± 56.75 | 206.03 ± 72.96 | 175.86 ± 35.60 |
| AUC_iv (ng∙h∙mL^-1^) | - | 682.16 ± 67.89 | 687.72 ± 36.56 |
| AUC_hpv (ng∙h∙mL^-1^) | - | 329.17 ± 47.90 | 329.16 ± 24.62 |

a: Median, instead of mean ±1 SD, is calculated for T_max_.
