# Supplementary material for: In silico drug absorption tract: An agent-based biomimetic model for human oral drug absorption
Source: PLoS One. 2018 Aug 31;13(8):e0203361. doi: 10.1371/journal.pone.0203361 (PMC6118387; doi:10.1371/journal.pone.0203361)
Supplement: S4 Table — (DOCX) [file pone.0203361.s011.docx]

S4 Table. PK parameters (Mean ±1 SD) of clonazepam (N=14)

| PK Parameters | Referent | Raw simulated | Smoothed ( ± 10 steps) simulated |
| --- | --- | --- | --- |
| AUC_po (ng∙h∙mL^-1^) | 529.08 ± 80.79 | 558.37 ± 168.65 | 523.71 ± 74.48 |
| C_max_ (ng∙mL^-1^) | 16.20 ± 4.28 | 22.68 ± 6.88 | 15.96 ± 3.30 |
| T_max_ (h) ^a^ | 1.50 | 3.875 | 1.50 |
| Kel (h^-1^) | 0.021 ± 0.004 | 0.023 ± 0.011 | 0.018 ± 0.005 |
| T_1/2_ (h) | 49.02 ± 7.79 | 57.03 ± 32.53 | 60.86 ± 18.12 |
| CL/F (L∙h^-1^) | 3.88 ± 0.68 | 3.91 ± 1.28 | 3.89 ± 0.55 |
| V/F (L) | 186.84 ± 25.25 | 221.22 ± 144.74 | 230.06 ± 46.81 |
| AUC_iv (ng∙h∙mL^-1^) | - | 646.15 ± 150.82 | 657.64 ± 93.20 |
| AUC_hpv (ng∙h∙mL^-1^) | - | 632.69 ± 133.44 | 574.73 ± 56.62 |

a: Median, instead of mean ±1 SD, is calculated for T_max_.
