# Supplementary material for: In silico drug absorption tract: An agent-based biomimetic model for human oral drug absorption
Source: PLoS One. 2018 Aug 31;13(8):e0203361. doi: 10.1371/journal.pone.0203361 (PMC6118387; doi:10.1371/journal.pone.0203361)
Supplement: S5 Table — (DOCX) [file pone.0203361.s012.docx]

S5 Table. PK parameters (Mean ±1 SD) of midazolam at speculated scenarios (N=15)

| PK Parameters | Simulated ^a^ | Simulated ^b^ | Simulated ^c^ | Simulated ^d^ |
| --- | --- | --- | --- | --- |
| AUC_po (ng∙h∙mL^-1^) | 318.83 ± 31.01 | 309.76 ± 30.61 | 204.92 ± 25.20 | 512.16 ± 36.69 |
| C_max_ (ng∙mL^-1^) | 77.81 ± 7.30 | 71.84 ± 8.82 | 75.38 ± 9.53 | 81.28 ± 12.70 |
| T_max_ (h) ^e^ | 1.00 | 1.50 | 1.00 | 1.00 |
| Kel (h^-1^) | 0.28 ± 0.04 | 0.27 ± 0.04 | 0.53 ± 0.08 | 0.14 ± 0.02 |
| T_1/2_ (h) | 3.71 ± 0.66 | 3.74 ± 0.63 | 1.93 ± 0.34 | 1.95 ± 0.86 |
| CL/F (L∙h^-1^) | 47.47 ± 4.72 | 48.86 ± 4.76 | 74.28 ± 9.47 | 29.42 ± 2.06 |
| V/F (L) | 175.86 ± 35.60 | 182.56 ± 33.83 | 142.02 ± 21.36 | 204.26 ± 26.80 |

All the PK parameters are calculated based on smoothed (±10 steps) simulated data.

a: Baseline values.

b: Retarded stomach flow by setting flowrate in stomach to 1%.

c: Enhanced CYP activity by setting metabolizeProb to 200%.

d: Reduced hepatic CYP amount by setting numcyp to 50%.

e: Median of T_max_ is calculated instead of Mean ±1 SD.
