# Supplementary material for: In silico drug absorption tract: An agent-based biomimetic model for human oral drug absorption
Source: PLoS One. 2018 Aug 31;13(8):e0203361. doi: 10.1371/journal.pone.0203361 (PMC6118387; doi:10.1371/journal.pone.0203361)
Supplement: S6 Table — (DOCX) [file pone.0203361.s013.docx]

S6 Table. PK parameters (Mean ±1 SD) of clonazepam at speculated scenarios (N=14)

| PK Parameters | Simulated ^a^ | Simulated ^b^ | Simulated ^c^ | Simulated ^d^ |
| --- | --- | --- | --- | --- |
| AUC_po (ng∙h∙mL^-1^) | 523.71 ± 74.48 | 543.52 ± 66.93 | 378.62 ± 83.14 | 709.89 ± 99.41 |
| C_max_ (ng∙mL^-1^) | 15.96 ± 3.30 | 15.12 ± 2.82 | 15.67 ± 2.16 | 15.49 ± 1.43 |
| T_max_ (h) ^e^ | 1.50 | 1.00 | 1.25 | 1.00 |
| Kel (h^-1^) | 0.018 ± 0.005 | 0.017 ± 0.004 | 0.034 ± 0.009 | 0.01 ±0.005 |
| T_1/2_ (h) | 60.86 ± 18.12 | 63.52 ± 19.11 | 31.93 ± 9.87 | 156.97 ± 96.46 |
| CL/F (L∙h^-1^) | 3.89 ± 0.55 | 3.73 ± 0.45 | 5.53 ± 1.22 | 2.87 ± 0.43 |
| V/F (L) | 230.06 ± 46.81 | 234.31 ± 64.50 | 171.32 ± 48.89 | 425.75 ± 222.20 |

All the PK parameters are calculated based on smoothed (±10 steps) simulated data.

a: Baseline values.

b: Retarded stomach flow by setting flowrate in stomach to 1%.

c: Enhanced CYP activity by setting metabolizeProb to 200%.

d: Reduced hepatic CYP amount by setting numcyp to 50%.

e: Median of T_max_ is calculated instead of Mean ±1 SD.
